# The association between diabetes knowledge, treatment satisfaction and medication adherence among Malaysian geriatric patients

**DOI:** 10.1007/s13340-025-00860-8

**Published:** 2025-11-25

**Authors:** Nur Syaqirah Natasha Mohamad Yusaini, Muhammad Eid Akkawi

**Affiliations:** 1 Department of Pharmacy Practice, Faculty of Pharmacy, International Islamic University Malaysia, Kuantan, Malaysia; 2 Quality Use of Medicine Research Group, Faculty of Pharmacy, International Islamic University Malaysia, Jalan Sultan Ahmad Shah, Indera Mahkota, 25200 Kuantan, Pahang Malaysia

**Keywords:** Diabetes knowledge, Treatment satisfaction, Medication adherence, Older adults, Treatment outcomes, Malaysia

## Abstract

**Background:**

Medication adherence among geriatric diabetic patients is influenced by various factors, including diabetes knowledge and treatment satisfaction. Understanding these relationships is crucial for improving adherence and health outcomes.

**Methods:**

A cross-sectional study was conducted among 300 diabetic patients aged 60 and above at outpatient clinics of a Malaysian teaching hospital. Interviews were conducted for each participant using a set of questionnaires that included a sociodemographic form, 20 questions from the simplified Diabetes Knowledge Test (DKT), 11 questions from the Treatment Satisfaction Questionnaire for Medication (TSQM-II), and 12 questions from the Malaysia Medication Adherence Assessment Tool (MyMAAT).

**Results:**

Participants demonstrated moderate diabetes knowledge [median = 6.67(6.00–7.78)] and high medication adherence [73%]. Diabetes knowledge was significantly associated with age [70–79 years: p = 0.012, above 80: *p* = 0.007], educational status [high school: *p* = 0.007, college/university: *p* < 0.001], and medication type [the presence of insulin in the regimen: *p* = 0.009]. A significant relationship was found between diabetes knowledge and treatment satisfaction [*p* < .001] and medication adherence [*p* = 0.004]. Each one-unit increase in diabetes knowledge was associated with a 34.2% decrease in the odds of nonadherence (OR = 0.658, 95% CI: 0.494–0.876, *p* = 0.004). Factors like gender [female: *p* = 0.014], occupational status [retired/ unemployed: *p* = 0.022], and type of diabetes medications [*p* < .001] influenced treatment satisfaction, while education [high school: *p* = 0.004] and global satisfaction [*p* = 0.009] affected adherence.

**Conclusions:**

Geriatric diabetic patients demonstrated inadequate knowledge about diabetes, and this limited knowledge was significantly associated with lower treatment satisfaction and poorer medication adherence.

## Introduction

Diabetes mellitus is a major non-communicable disease that poses significant public health and economic challenges worldwide [1]. Its prevalence is expected to rise from 422 million cases globally to 693 million by 2045 [2]. In Malaysia, this trend is particularly evident. The National Health and Morbidity Survey (NHMS) 2019 reported a high diabetes prevalence of 41.5% among Malaysians aged 60 and above which was 34.4% in 2011 [3, 4].

Studies indicate that 60% of older Malaysian adults do not take their medications as directed and 46% of diabetic patients aged 18 and above show nonadherence [5, 6]. This is concerning, as older diabetic patients face higher risks of macrovascular and microvascular complications. In fact, nonadherence is linked to poorer health outcomes, higher hospitalization rates, longer hospital stays, and increased medical costs [7, 8].

Effective diabetes management relies on medication adherence and understanding the disease. Knowledge about diabetes is crucial for patients to appreciate the importance of controlling blood glucose and preventing complications [9] Patients with insufficient knowledge about their disease usually demonstrate lower adherence to medication regimens which can lead to negative health outcomes [10]. Despite this, studies in Malaysia reported low diabetes knowledge, ranging from 33.6 to 73.5% [11].

Understanding diabetes is important for taking medication, but being satisfied with treatment also helps patients stick to their plans. Research demonstrates that higher treatment satisfaction leads to better medication adherence and is associated with improved blood glucose control [12, 13]. Structured health education, insulin therapy for blood glucose control, and advanced digital treatments can enhance the patient experience, suggesting that treatment satisfaction is linked to diabetes knowledge [14].

As highlighted above, diabetes is quite prevalent among older adults in Malaysia while their diabetes knowledge and medication adherence were reported to be poor. Although, previous research highlights the importance of diabetes knowledge, limited studies have investigated its effect on medication adherence and treatment satisfaction in Malaysia’s older population. This study aims to examine the relationships between diabetes knowledge, treatment satisfaction, and medication adherence among older diabetic outpatients, providing insights to improve diabetes management in this group.

## Methods

### Study designs and settings

This cross-sectional study involved older diabetic patients attending outpatient clinics and the outpatient pharmacy at Sultan Ahmad Shah Medical Centre (SASMEC), a teaching hospital located in Kuantan, Malaysia.

### Sample size

The required sample size was calculated using the single-proportion formula, based on a 95% confidence interval (CI), a 5% margin of error, and a prevalence of low medication adherence among patients with diabetes in Malaysia of 24.0% [15]. The formula used is as below:

$$ n = \frac{{Z^{2} \cdot p \cdot \left( {1 - p} \right)}}{{E^{2} }} $$

where n is the required sample size, Z is the z-score for 95% *CI* (Z = 1.96) p is the prevalence (*p* = 0.24), and E is the margin of error. The calculation suggested a minimum sample size of 280 older patients.

### Study population

Participants were included if they:

- were at least 60 years old,
- had type-2 diabetes and had been on medication for at least 3 months, and
- could communicate in Malay and provide consent.

Patients with cognitive impairments or psychological disorders were excluded.

### Data collection

Data were collected through direct interviews and review of the electronic medical records, covering sociodemographic, comorbidities (Charlson Comorbidity Index, CCI), and medication information. Participants were also asked to complete the Simplified Diabetes Knowledge Test (DKT), the Treatment Satisfaction Questionnaire for Medication (TSQM), and the Malaysia Medication Adherence Assessment Tool (MyMAAT).

#### Charlson comorbidity index (CCI)

The CCI was created to help predict long-term mortality based on various medical conditions a person may have. It includes specific comorbidities with weights assigned according to how much they affect mortality risk. A score of 0 indicates no comorbidities, 1–2 suggests mild comorbidities, 3–4 indicates moderate comorbidities and 5 or higher points to severe comorbidities [16].

#### Simplified version of diabetes knowledge test (DKT)

Diabetes knowledge was assessed using the simplified Diabetes Knowledge Test (DKT), comprising 20 true–false questions. Insulin users should answer all 20 questions, while non-insulin users answer 18 questions [17]. Each correct answer was awarded 1 point, and the total score was prorated to a 10-point scale to ensure consistency in comparison regardless of the number of questions answered. The DKT was previously translated to Malay language, and it was validated among low-literacy older patients [18].

#### Treatment satisfaction questionnaire for medication (TSQM-II)

The 11-item Malay version of the Treatment Satisfaction Questionnaire for Medication (TSQM-II) was used to assess medication satisfaction across four domains: effectiveness, side effects, convenience, and global satisfaction. Scores were calculated using the scale’s scoring algorithm with results ranging from 0 to 100 for each domain [19]. The TSQM-II questionnaire was translated to Malay language and validated among Malaysian older adults. The Malay version of TSQM-II was found to be valid, reliable and psychometrically sound for assessing treatment satisfaction among Malay-speaking populations. [20].

#### Malaysia medication adherence assessment tool (MyMAAT)

Medication adherence was assessed with the 12-item MyMAAT, with responses ranging from 1 to 5 points for each question. A score between 12–53 indicates nonadherence, while 54–60 indicates adherence [21]. The questionnaire was originally developed in Malay language and validated. The questionnaire demonstrated excellent psychometric properties, with strong internal consistency (Cronbach’s alpha = 0.91) and high test–retest reliability (intraclass correlation coefficient = 0.97) [21].

### Statistical analysis

All statistical analyses were conducted using Jamovi for Windows (Version 2.6.13). Descriptive statistics summarized the data, with continuous variables reported as medians (IQR) and categorical variables as frequencies and percentages. Outliers were checked, and normality was assessed using the Shapiro–Wilk test. To examine the relationship, Kruskal–Wallis and Mann–Whitney tests were used for non-normally distributed variables. The Chi-square test analyzed medication adherence by categorical variables. Spearman’s correlation assessed the relationship between diabetes knowledge and treatment satisfaction. Multivariate linear regression and logistic regression models were applied to control for confounders with a p-value < 0.25. All assumptions were verified before analysis.

## Results

### Association between sociodemographic and diabetes knowledge

The study included 300 older adults with diabetes, primarily aged 60–69 (67.7%), with a nearly equal gender distribution (50.3% male, 49.7% female). Additionally, most lived with others (96%) and were retired or unemployed (95.3%). The majority had low household incomes (62%) and high school education backgrounds (45.3%). Nearly half had diabetes for over 10 years (49%), and notably, most of them were not using insulin (58.3%). Moreover, many had severe comorbidities (56%), with an average of 7 medications per patient (Table 1).

**Table 1:** Association between demographic characteristics and diabetes Knowledge: Bivariate and Multivariate Analyses (N: 300)

		Bivariate analysis		Multivariate analysis		
Variables	Patients N (%)	Diabetes Knowledge [Median (IQR)]	*p*–value	β	95% CI	*p*–value
All Patients		6.67 (6.00–7.78)				
Age 60–69 years 70–79 years above 80 years	203 (67.7%) 83 (27.7%) 14 (4.7%)	7.00 (6.11–7.78) 6.50 (5.50–7.22) 6.11 (5.00–6.67)	< .001^a^	1 –0.40303 –0.90861	Ref [− 0.715, − 0.0910] [− 1.561, − 0.2562]	Ref 0.012 0.007
Gender Male Female	151 (50.3%) 149 (49.7%)	6.67 (6.00–7.78) 6.67 (6.00–7.50)	0.572^b^	–	–	–
Living arrangement Living alone Not living alone	12 (4.0%) 288 (96.0%)	6.50 (5.00–6.75) 6.67 (6.00–7.78)	0.148^b^	1 –0.14373	Ref [− 0.775, 0.4880]	Ref 0.655
Occupational status Employed Retired/ Unemployed	14 (4.7%) 286 (95.3%)	7.22 (6.75–8.19) 6.67 (6.00–7.50)	0.119^b^	1 0.28383	Ref [− 0.375, 0.9427]	Ref 0.397
Household income < RM2500 RM2500–RM5000 > RM5000	186 (62.0%) 83 (27.7%) 31 (10.3%)	6.50 (5.56–7.22) 7.22 (6.30–7.78) 7.50 (6.50–8.16)	< .001^a^	1 0.05738 0.15957	Ref [− 0.268, 0.3829] [− 0.332, 0.6509]	Ref 0.729 0.523
Educational status No formal education/ Primary school High school College/ University	75 (25.0%) 136 (45.3%) 89 (29.7%)	6.11 (5.00–6.67) 6.67 (6.00–7.50) 7.50 (6.67–7.78)	< .001^a^	1 0.47519 1.10204	Ref [0.132, 0.8188] [0.696, 1.5077]	Ref 0.007 < .001
Duration of diabetes Less than 5 years 5 to 10 years More than 10 years	82 (27.3%) 71 (23.7%) 147 (49.0%)	6.83 (6.11–7.78) 6.67 (6.11–7.50) 6.67 (6.00–7.50)	0.464^a^	–	–	–
Medications for diabetes OHA only Using Insulin	175 (58.3%) 119 (41.7%)	7.22 (6.11–7.78) 6.50 (5.50–7.00)	< .001^b^	1 –0.35660	Ref [− 0.622, − 0.0909]	Ref 0.009
CCI 3–4 (Moderate comorbidities) ≥ 5 (Severe comorbidities)	132 (44.0%) 168 (56.0%)	7.00 (6.11–7.78) 6.67 (5.50–7.50)	0.005^b^	1 0.00347	Ref [− 0.301, 0.3077]	Ref 0.982
Number of medications 1–4 5–9 Above 9	[7.00 (5.00–9.00)] 36 (12.0%) 207 (69.0%) 57 (19.0%)	7.22 (6.50–7.78) 6.67 (6.00–7.78) 6.50 (5.50–7.22)	0.005^a^	1–0.25093 –0.51774	Ref [− 0.665, 0.1628] [− 1.048, 0.0130]	Ref 0.234 0.056

The median diabetes knowledge score across all participants was 6.67 (IQR: 6.00–7.78). Bivariate analysis showed significant differences in diabetes knowledge scores between groups defined by age, household income, educational status, medication type, CCI, and the number of medications taken (*p* < 0.05). This indicates that these variables are associated with variations in diabetes knowledge (Table 1).

In the multivariate analysis, we included factors like age, living arrangement, occupational status, household income, educational status, diabetes medication, CCI, and number of medications due to their *p*-values < 0.25 in the bivariate analysis. Results showed that only three factors remained significant, Table 1. Firstly, age was significantly associated with diabetes knowledge, with individuals aged 70–79 years having lower knowledge scores than those aged 60–69 (*β* = -0.40303, 95%CI [− 0.715, − 0.0910], *p* = 0.012). Similarly, those above 80 years had even lower knowledge scores (*β* = − 0.90861, 95%CI [− 1.561, − 0.2562], *p* = 0.007), indicating a decline in diabetes knowledge with increasing age. Educational status was also a significant factor, with individuals who completed high school having higher diabetes knowledge scores than those with only primary school education (*β* = 0.47519, 95%CI [0.132, 0.8188], *p* = 0.007). Moreover, those with college/ university education had significantly higher knowledge scores (*β* = 1.10204, 95% *CI* [0.696, 1.5077], *p* < 0.001). Medication type was another significant factor in the analysis. The results indicate that individuals who have insulin as part of their regimens have lower diabetes knowledge scores compared to those using OHA only (*β* = − 0.35660, 95%CI [− 0.622, − 0.0909], *p* = 0.009).

### Treatment satisfaction and associated factors

As shown in Table 2, participants generally reported high satisfaction with side effects [100] and convenience [83.3 (IQR: 72.2–94.4)] among the treatment satisfaction domains. Notably, only 40 participants reported experiencing side effects which contributed to the extremely high median score in this domain. Several factors were significantly associated with treatment satisfaction. Age was significantly associated with effectiveness with younger older patients (60–79 years) reporting higher scores in the effectiveness domain than those above 80 (*p* = 0.016). In addition, gender differences were noted especially in effectiveness (*p* = 0.001) and global satisfaction (*p* = 0.002). Also, educational status played a role in treatment satisfaction, significantly influencing effectiveness (*p* = 0.042), convenience (*p* = 0.003), and global satisfaction (*p* = 0.024). Furthermore, medication type was linked to convenience (*p* < 0.001) and global satisfaction (*p* = 0.032), while the number of medications taken was significantly associated with convenience only (*p* = 0.031).

**Table 2:** Association between demographic characteristics and domains of treatment satisfaction (N:300)

Variables	Treatment satisfaction [Median (IQR), Mean ± SD]			
	Effectiveness	Side effects	Convenience	Global satisfaction
All patients	66.7 (66.7–83.3) 71.5 ± 14.8	100 98.3 ± 4.6	83.3 (72.2–94.4) 83.1 ± 13.9	75.0 (66.7–83.3) 75.1 ± 12.6
Age 60–69 years 70–79 years above 80 years	66.7 (66.7–83.3) 72.5 ± 1.1 66.7 (66.7–83.3) 70.8 ± 1.5 66.7 (58.3–66.7) 70 ± 2.7	100 98.1 ± 0.3 100 98.5 ± 0.5 100 100 ± 0.1	83.3 (72.2–100) 83.4 ± 1.0 83.3 (72.2–91.7) 81.3 ± 1.5 83.3 (73.6–87.5) 82.5 ± 3.1	75.0 (66.7–83.3) 75.5 ± 0.9 75.0 (66.7–83.3) 74.7 ± 1.4 66.7 (66.7–7.50) 69.6 ± 2.6
*p–value*	0.016^a^	0.248^a^	0.337^a^	0.228^a^
Gender Male Female	66.7 (66.7–83.3) 74.3 ± 1.2 66.7 (66.7–75.0) 68.8 ± 1.2	100 98.3 ± 0.4 100 98.2 ± 0.4	83.3 (77.8–100) 84.5 ± 1.1 83.3 (72.2–94.4) 81.6 ± 1.2	75.0 (66.7–83.3) 77.3 ± 1.0 75.0 (66.7–83.3) 72.8 ± 1.0
*p–value*	0.001^b^	0.834^b^	0.053^b^	0.002^b^
Living arrangement Living alone Not living alone	66.7 (64.6–77.1) 72.3 ± 4.3 66.7 (66.7–83.3) 71.5 ± 0.9	100 97.9 ± 1.5 100 98.3 ± 0.3	83.3 (70.8–88.9) 81.5 ± 3.6 83.3 (77.2–94.4) 83.1 ± 0.8	70.8 (64.6–77.1) 73.6 ± 4.2 75.0 (66.7–83.3) 75.1 ± 0.7
*p–value*	0.907^b^	0.800^b^	0.632^b^	0.460^b^
Occupational status Employed Retired/ Unemployed	70.8 (66.7–83.3) 75.6 ± 2.8 66.7 (66.7–83.3) 71.3 ± 0.9	100 (93.8–100) 97.0 ± 1.4 100 98.4 ± 0.3	80.6 (66.7–91.7) 79.0 ± 4.0 83.3 (72.2–94.4) 83.3 ± 0.8	70.8 (66.7–75.0) 73.2 ± 2.8 75.0 (66.7–83.3) 75.1 ± 0.7
*p–value*	0.156^b^	0.137^b^	0.222^b^	0.463^b^
Household income < RM2500 RM2500–RM5000 > RM5000	66.7 (66.7–83.3) 70.1 ± 1.0 66.7 (66.7–83.3) 73.3 ± 1.8 66.7 (66.7–83.3) 75.3 ± 2.6	100 98.5 ± 0.3 100 97.9 ± 0.6 100 97.8 ± 0.9	83.3 (72.2–94.4) 83.2 ± 1.0 83.3 (77.8–97.2) 84.3 ± 1.5 83.3 (66.7–94.4) 79.2 ± 3.1	75.0 (66.7–83.3) 74.9 ± 0.9 75.0 (66.7–83.3) 75.7 ± 1.5 75.0 (66.7–83.3) 74.2 ± 2.3
*p–value*	0.078^a^	0.306^a^	0.360^a^	0.854^a^
Educational status No formal education/ Primary school High school College/ University	66.7 (58.3–83.3) 68.0 ± 1.7 66.7 (66.7–83.3) 72.7 ± 1.2 66.7 (66.7–83.3) 72.7 ± 1.6	100 98.0 ± 0.6 100 98.7 ± 0.3 100 97.8 ± 0.5	77.8 (69.4–88.9) 79.0 1.4 86.1 (77.8–100) 84.9 ± 1.1 83.3 (72.2–100) 83.8 ± 1.6	66.7 (66.7–83.3) 71.8 ± 1.4 75.0 (66.7–83.3) 76.6 ± 1.0 75.0 (66.7–83.3) 75.3 ± 1.4
*p–value*	0.042^a^	0.300^a^	0.003^a^	0.024^a^
Duration of diabetes Less than 5 years 5 to 10 years More than 10 years	66.7 (66.7–83.3) 71.6 ± 1.5 66.7 (66.7–83.3) 71.1 ± 2.0 66.7 (66.7–83.3) 71.7 ± 1.2	100 98.1 ± 0.5 100 98.5 ± 0.5 100 98.2 ± 0.4	88.9 (77.8–100) 85.8 ± 1.6 83.3 (77.8–91.7) 83.0 ± 1.6 83.3 (72.2–94.4) 81.6 ± 1.1	75.0 (66.7–83.3) 75.0 ± 1.2 75.0 (66.7–83.3) 75.0 ± 1.7 75.0 (66.7–83.3) 75.1 ± 1.0
*p–value*	0.929^a^	0.916^a^	0.051^a^	1.000^a^
Medications for diabetes Oral antihyperglycemic agent (OHA) only OHA & Insulin/ Insulin only	66.7 (66.7–83.3) 72.3 ± 1.1 66.7 (66.7–83.3) 70.4 ± 1.3	100 98.5 ± 0.3 100 98.0 ± 0.5	88.9 (77.8–100) 86.6 ± 0.9 77.8 (66.7–88.9) 78.2 ± 1.3	75.0 (66.7–83.3) 76.3 ± 0.9 75.0 (66.7–83.3) 73.1 ± 1.1
*p–value*	0.283^b^	0.648^b^	< .001^b^	0.032^b^
CCI 3–4 (Moderate comorbidities) ≥ 5 (Severe comorbidities)	66.7 (66.7–83.3) 71.3 ± 1.3 66.7 (66.7–83.3) 71.7 ± 1.1	100 97.7 ± 0.5 100 98.7 ± 0.3	83.3 (76.4–100) 84.1 ± 1.2 83.3 (72.2–94.4) 82.2 ± 1.1	75.0 (66.7–83.3) 74.9 ± 1.1 75.0 (66.7–83.3) 75.1 ± 1.0
*p–value*	0.842^b^	0.084^b^	0.365^b^	0.999^b^
Number of medications [7.00 (5.00–9.00)] 1–4 5–9 Above 9	66.7 (66.7–75.0) 70.1 ± 1.9 66.7 (66.7–83.3) 71.6 ± 1.1 66.7 (66.7–83.3) 71.9 ± 1.8	100 96.7 ± 1.1 100 98.4 ± 0.3 100 98.7 ± 0.5	83.3 (77.8–100) 87.0 ± 1.9 83.3 (72.2–94.4) 83.6 ± 0.9 83.3 (66.7–88.9) 78.5 ± 2.1	75.0 (66.7–77.1) 75.0 ± 1.6 75.0 (66.7–83.3) 74.9 ± 0.9 75.0 (66.7–83.3) 75.3 ± 1.7
*p–value*	0.763^a^	0.120^a^	0.031^a^	0.985^a^

^a^Using Kruskal–Wallis; ^b^Using Mann–Whitney

CCI: charlson comorbidity index

A Spearman correlation was conducted to examine the relationship between diabetes knowledge and the treatment satisfaction domains, Table 3. A significant positive correlation was observed between diabetes knowledge and the effectiveness domain of treatment satisfaction (*r* = 0.129, *p* = 0.025), as well as the convenience domain (*r* = 0.257, *p* < 0.001). Conversely, there was a significant negative correlation between diabetes knowledge and the side effects domain (*r* = − 0.156, *p* = 0.007). Lastly, a small but significant positive correlation was found between diabetes knowledge and global satisfaction (*r* = 0.122, *p* = 0.034).

**Table 3:** Correlation between diabetes knowledge and treatment satisfaction (N: 300)

	Bivariate analysis		Multivariate analysis
	Correlation coefficient (r)	*p*–value	*p*–value
Effectiveness	0.129	0.025	0.325
Side effects	− 0.156	0.007	–
Convenience	0.257	< .001	< .001
Global Satisfaction	0.122	0.034	0.211

Only three domains were analyzed using multivariate analysis to assess the independent contributions of diabetes knowledge to treatment satisfaction while controlling for potential confounders including age, living arrangement, occupational status, household income, educational status, type of diabetes medications, CCI and number of medications. The side effects domain was excluded because only 13.33% of participants reported side effects, making the data less reliable. Confounding factors were selected based on a *p* < 0.25 from the bivariate analysis (Table 2). However, only the convenience domain showed a significant relationship with diabetes knowledge, Table 3.

Table 4 shows that diabetes knowledge was significantly and positively associated with convenience (*β* = 2.0306, 95% *CI* [1.01, 3.604], *p* < 0.001). This reflects that for every one-unit increase in diabetes knowledge, the convenience score increases by approximately 2.0306 units, holding all other variables constant. Gender was a significant factor, with females reporting lower convenience score compared to males (*β* = − 3.880, 95% *CI* [− 6.97, − 0.792], *p* = 0.014). The occupational status also had a significant impact, where retired or unemployed individuals had higher convenience score compared to employed individuals (*β* = 8.202, 95% *CI* [1.20, 15.201], *p* = 0.022). Type of diabetes medications was a significant factor, with patients using both OHA and insulin reporting significantly lower convenience score compared to those using OHA only (*β* = − 7.275, 95% *CI* [− 10.41, − 4.142], *p* < 0.001).

**Table 4:** The multivariate linear regression analysis of factors influencing convenience domain of the treatment satisfaction (N: 300)

Variables	β	95% CI	*p*–value
Diabetes knowledge	2.0306	[1.01, 3.604]	< .001
Gender Male Female	1 – 3.880	Ref [– 6.97, – 0.792]	Ref 0.014
Occupational status Employed Retired/ Unemployed	1 8.202	Ref [1.20, 15.201]	Ref 0.022
Educational status No formal education/ Primary school High school College/ University	1 2.764 – 1.075	Ref [– 1.14, 6.664] [– 5.66, 3.509]	Ref 0.164 0.645
Duration of diabetes Less than 5 years 5 to 10 years More than 10 years	1 – 0.500 – 0.707	Ref [– 4.65, 3.646] [– 4.38, 2.962]	Ref 0.812 0.705
Medications for diabetes Oral antihyperglycemic agent (OHA) only OHA & Insulin/ Insulin only	1 – 7.275	Ref [– 10.41, – 4.142]	Ref < .001
Number of medications 1–4 5–9 Above 9	1 – 1.838 – 4.823	Ref [– 6.48, 2.807] [– 10.41, 0.767]	Ref 0.437 0.091

*R* = 0.430; Adjusted *R*^*2*^ = 0.156

### Association of diabetes knowledge, treatment satisfaction, and other factors with medication adherence

Table 5 depicts that 73% of patients were adherent to their treatment. Educational status was significantly associated with adherence (*p* < 0.001), with a higher proportion of nonadherence among those with no formal education/primary school education (48%) compared to high school (19.9%) and college/university education (20.2%). Medication adherence was also significantly associated with the type of diabetes medications (*p* = 0.007). Patients on OHA only had higher adherence (78.9%) compared to those on a combination of OHA and insulin or insulin alone (64.8%). No significant associations were found between adherence and age, gender, living arrangement, occupational status, household income, duration of diabetes, CCI, or number of medications (*p* > 0.05 for all).

**Table 5:** Association between demographic characteristics and medication adherence (N: 300)

Variables	Medication adherence		
	Adherence N (%)	Nonadherence	*p*–value^d^
	219 (73)	81 (27)	
Age 60–69 years 70–79 years above 80 years	152 (69.4) 60 (27.4) 7 (3.2)	51 (63) 23 (28.4) 7 (8.6)	0.126
Gender Male Female	115 (52.5) 104 (47.5)	36 (44.4) 45 (55.6)	0.215
Living arrangement Living alone Not living alone	11 (5.0) 208 (95.0)	1 (1.2) 80 (98.8)	0.137
Occupational status Employed Retired/ Unemployed	10 (4.6) 209 (95.4)	4 (4.9) 77 (95.1)	0.892
Household income < RM2500 RM2500–RM5000 > RM5000	131 (59.8) 64 (29.2) 24 (11.0)	55 (67.9) 19 (23.5) 7 (8.6)	0.440
Educational status No formal education/ Primary school High school College/ University	39 (17.8) 109 (49.8) 71 (32.4)	36 (44.4) 27 (33.3) 18 (22.2)	< .001
Duration of diabetes Less than 5 years 5 to 10 years More than 10 years	63 (28.8) 51 (23.3) 105 (47.9)	19 (23.5) 20 (24.7) 42 (51.9)	0.656
Medications for diabetes Oral antihyperglycemic agent (OHA) only OHA & Insulin/ Insulin only	138 (63) 81 (37)	37 (45.7) 44 (54.3)	0.007
CCI 3–4 (Moderate comorbidities) ≥ 5 (Severe comorbidities)	103 (47) 116 (53)	29 (35.8) 52 (64.2)	0.082
Number of medications 1–4 5–9 Above 9	31 (14.2) 143 (65.3) 45 (20.5)	5 (6.2) 64 (79.0) 12 (14.8)	0.056
Effectiveness	66.7 (66.7–83.3)	66.7 (50.0–66.7)	< .001^b^
Side effects	100	100	0.809^b^
Convenience	83.3 (77.8–100)	77.8 (66.7–83.3)	< .001^b^
Global satisfaction	75.0 (66.7–83.3)	66.7 (58.3–75.0)	< .001^b^

^b^ Using Mann–Whitney; ^d^ Using Chi–square

CCI: charlson comorbidity index

In addition, treatment satisfaction differed significantly between adherent and non-adherent patients. Those who adhered to their medication reported higher satisfaction in effectiveness [66.7(66.7–83.3) vs. 66.7(50.0–66.7), *p* < 0.001], convenience [83.3(77.8–100) vs. 77.8(66.7–83.3), *p* < 0.001], and global satisfaction [75.0(66.7–83.3) vs. 66.7(58.3–75.0), *p* < 0.001]. However, there was no significant difference in satisfaction related to side effects (*p* = 0.809).

To check whether diabetes knowledge would still impact medication adherence after controlling covariables, a multivariate logistic regression analysis was applied, Table 6. It included variables with significant associations and those with a *p* < 0.25, namely, age, gender, living arrangement, educational status, type of diabetes medications CCI, number of medications and treatment satisfaction domains. Diabetes knowledge remained a significant factor on medication adherence, where each one-unit increase in diabetes knowledge is associated with 34.2% decrease in the odds of nonadherence (OR = 0.658, 95% *CI* [0.494–0.876], *p* = 0.004). Compared to individuals with no formal or primary education, those with a high school education have 70% lower odds of nonadherence to medication (*OR* = 0.300, 95% *CI*: 0.133–0.676, *p* = 0.004). Additionally, among those three treatment satisfaction domains only global satisfaction was found to be significantly associated with adherence. The higher global satisfaction was significantly linked to a reduced likelihood of nonadherence, suggesting better adherence (*OR* = 0.948, 95% *CI* [0.911, 0.987], *p* = 0.009).

**Table 6:** Logistic regression analysis of the impact of diabetes knowledge and other factors on medication nonadherence (N: 300)

Variables	β	SE	*p*–value	OR	95% CI
Diabetes knowledge	− 0.4189	0.1464	0.004	0.658	[0.494, 0.876]
Age 60–69 years 70–79 years above 80 years	1 − 0.5451 − 0.2414	Ref 0.3934 0.7375	Ref 0.166 0.743	Ref 0.580 0.786	Ref [0.268, 1.254] [0.158, 3.333]
Gender Male Female	1 − 0.1602	Ref 0.3379	Ref 0.636	Ref 0.852	Ref [0.439, 1.652]
Living arrangement Living alone Not living alone	1 1.7185	Ref 1.1165	Ref 0.124	Ref 5.576	Ref [0.625, 49.742]
Educational status No formal education/ Primary school High school College/ University	1 − 1.2031 − 0.8591	Ref 0.4140 0.4761	Ref 0.004 0.071	Ref 0.300 0.424	Ref [0.133, 0.676] [0.167, 1.077]
Medications for diabetes Oral antihyperglycemic agent (OHA) only OHA & Insulin/ Insulin only	1 0.4173	Ref 0.3335	Ref 0.211	Ref 1.518	Ref [0.790, 2.918]
CCI 3–4 (Moderate comorbidities) ≥ 5 (Severe comorbidities)	1 0.4520	Ref 0.3648	Ref 0.215	Ref 1.571	Ref [0.769, 3.213]
Number of medications 1–4 5–9 Above 9	1 0.6120 − 0.7196	Ref 0.5545 0.7087	Ref 0.270 0.310	Ref 1.844 0.487	Ref [0.622, 5.468] [0.121, 1.953]
Effectiveness	− 0.0206	0.0157	0.190	0.980	[0.950, 1.010]
Convenience	− 0.0124	0.0131	0.342	0.988	[0.963, 1.013]
Global satisfaction	− 0.0532	0.0205	0.009	0.948	[0.911, 0.987]

McFadden’s *R*^*2*^ = 0.259. β represents the log odds of being categorized as "Nonadherence" compared to "Adherence". CCI: Charlson Comorbidity Index

## Discussion

This study evaluated the levels of diabetes knowledge, treatment satisfaction, and medication adherence among older diabetic patients and examined the factors influencing these variables. The results showed a moderate level of diabetes knowledge (median score: 6.67), like another study from Malaysia, where patients aged 45–65 demonstrated acceptable knowledge [22]. The moderate diabetes knowledge observed in the middle-aged group from that study raised concerns -when compared to our findings in older adults- that knowledge levels do not significantly improve with age. This trend suggests a stagnation in diabetes knowledge among Malaysians that hinders older adults’ ability to manage their condition effectively. Likewise, studies from Türkiye and Brazil reported low diabetes knowledge among older adults [23, 24]. This low knowledge can be partly attributed to cognitive decline, which often accompanies ageing and significantly impairs the ability to process and retain diabetes-related information [23]. Additionally, Amaral et al. noted that Brazilian older adults frequently demonstrate lower diabetes knowledge due to factors like educational background and socioeconomic challenges [25]. These findings align with ours, where we observed that individuals with lower education had poorer diabetes knowledge. A systematic review further supports this observation, showing that individuals with higher education levels are better equipped to access, evaluate, and utilise health information, which helps them acquire valuable health knowledge [26].

Our findings are consistent with another research conducted in Malaysia where insulin users scored relatively low in diabetes knowledge despite receiving education on injection techniques and glucose monitoring [27]. This suggests that the instructions may not effectively enhance overall diabetes knowledge possibly due to the complexity of insulin regimens hindering users’ ability to fully process the information. However, other studies reported that insulin users tend to have better diabetes knowledge, most likely due to more frequent healthcare interactions [25, 28].

Next, we found that diabetes knowledge was significantly linked to several treatment satisfaction domains, including effectiveness, side effects, convenience, and global satisfaction, based on the Spearman correlation. However, after adjusting for confounding factors, only the convenience domain remained significantly associated with diabetes knowledge. From our study, most participants reported being satisfied with the convenience of their treatment. A previous study from Malaysia suggested that increased knowledge improved self-care, reduced stress, and enhanced treatment receptivity. This builds trust in healthcare providers, boosts self-efficacy, and enhances social acceptance, making treatment more convenient and manageable [22]. Thus, the link between diabetes knowledge and satisfaction is not straightforward. The convenience did not fully explain treatment satisfaction, suggesting that other factors like patient expectations, social support, or access to healthcare might have played a bigger role.

Additionally, we found that patients using insulin reported lower convenience compared to those on OHA only. This aligns with a study from India, which showed that treatment satisfaction was higher among patients receiving metformin alone or in combination with other OHA rather than insulin [29] The researchers noted that self-administering insulin was challenging particularly due to injection procedures and dietary adjustments needed to prevent insulin-induced hypoglycemia. Likewise, our findings suggest that inadequate diabetes knowledge might further contribute to these difficulties, which is a factor not emphasized in the Indian study. Also, older adults often face additional barriers to insulin use due to physical limitations including decreased dexterity, impaired vision, and physical capacity that can impede the self-injection process [30]

Our results depicted that diabetes knowledge played a key role in medication adherence, with most participants demonstrating high adherence to their prescribed medications. Like studies in Saudi Arabia and Malaysia, we found that patients with greater diabetes knowledge were more likely to adhere to their medications [31, 32]. Better diabetes education helps patients understand their condition and the importance of taking their medications which in turn leads to better glycemic control and fewer complications [33]. Education level was another critical factor influencing adherence where high school graduates were significantly more likely to adhere to their medication compared to those with only primary education. The Saudi study also found that higher education levels were associated with better medication adherence which aligns with our findings [31]. Additionally, prior studies from India and Nepal showed that individuals with higher education are better equipped to understand complex medical information and the importance of following their treatment plans [34, 35].

Another key factor influencing adherence was global satisfaction with treatment. Patients who were more satisfied with their treatment were more likely to be adherent. According to the TSQM-II framework, global satisfaction reflects a patient’s overall experience with their medication, including its effectiveness, side effects, and convenience [19]. This measure provides a holistic view of the patient's experience and offers a comprehensive evaluation of their satisfaction. Earlier studies from Palestine and Egypt stated that treatment satisfaction was positively associated with medication adherence which aligns with our study [12, 36]. Patients are more likely to take their prescribed medications as directed when they feel that their treatment is working, have a strong emotional bond with it, and see optimal clinical results [12]. On top of that, other Malaysian studies reported findings consistent with ours, highlighting that greater treatment satisfaction was linked to better adherence [13, 37].

## Limitations

The findings’ generalizability is limited because the sample was drawn from a single place, making it difficult to apply the results to the broader population of older diabetic patients in Malaysia. The study is also limited by its cross-sectional design, which precludes establishing causal relationships between diabetes knowledge and treatment satisfaction or adherence. Additionally, data about clinical outcomes such as glycemic control parameters and diabetes complications were not available which may affect the observed associations between the variables. Besides, although we adjusted for several demographic and clinical factors, the potential for residual confounding by unmeasured variables (such as cognitive function or access to healthcare facilities) cannot be excluded.

## Conclusion

This study found that geriatric diabetic patients have inadequate knowledge about diabetes. It also highlights the presence of a significant relationship between diabetes knowledge, treatment satisfaction, and medication adherence among this population. Also, it was found that patients who use insulin have lower diabetes knowledge than those using OHAs only. Therefore, enhancing diabetes education, especially for those on complex regimens like insulin, is a key to improving patient-reported outcomes. A comprehensive approach that prioritizes both knowledge and satisfaction can ultimately enhance diabetes care and quality of life among older adults. 

## Data Availability

Data are available upon request from the corresponding authors.
